# Supplementary material for: Household medication safety practices during the COVID-19 pandemic: a descriptive qualitative study protocol
Source: BMJ Open. 2020 Nov 24;10(11):e044441. doi: 10.1136/bmjopen-2020-044441 (PMC7688439; doi:10.1136/bmjopen-2020-044441)
Supplement: Author's manuscript [file bmjopen-2020-044441.draft_revisions.pdf]

BMJ Open is committed to open peer review. As part of this commitment we make the peer review history of every article we publish publicly available.

When an article is published we post the peer reviewers' comments and the authors' responses online. We also post the versions of the paper that were used during peer review. These are the versions that the peer review comments apply to.

The versions of the paper that follow are the versions that were submitted during the peer review process. They are not the versions of record or the final published versions. They should not be cited or distributed as the published version of this manuscript.

BMJ Open is an open access journal and the full, final, typeset and author-corrected version of record of the manuscript is available on our site with no access controls, subscription charges or pay-per-view fees (http://bmjopen.bmj.com).

If you have any questions on BMJ Open's open peer review process please email info.bmjopen@bmj.com

BMJ Open

Household Medication Safety during the Covid-19 Pandemic: A Descriptive Qualitative Study Protocol

Journal:	*BMJ Open*
Manuscript ID	bmjopen-2020-044441
Article Type:	Protocol
Date Submitted by the Author:	02-Sep-2020
Complete List of Authors:	Grimes, Tamasine C.; Trinity College, School of Pharmacy<br>Garfield, Sara; University College London, School of Pharmacy<br>Kelly, Dervla ; University of Limerick, Graduate Entry Medical School<br>Cahill, Joan; Trinity College Dublin, Centre for Innovative Human Studies<br>Cromie, Sam; Trinity College Dublin, Centre for Innovative Human Studies<br>Wheeler, Carly; Imperial College Healthcare NHS Trust, Centre for Medication Safety and Service Quality<br>Dean Franklin, Bryony; University College London School of Pharmacy,
Keywords:	Public health < INFECTIOUS DISEASES, COVID-19, PRIMARY CARE, QUALITATIVE RESEARCH, THERAPEUTICS

SCHOLARONE™
Manuscripts

I, the Submitting Author has the right to grant and does grant on behalf of all authors of the Work (as defined in the below author licence), an exclusive licence and/or a non-exclusive licence for contributions from authors who are: i) UK Crown employees; ii) where BMJ has agreed a CC-BY licence shall apply, and/or iii) in accordance with the terms applicable for US Federal Government officers or employees acting as part of their official duties; on a worldwide, perpetual, irrevocable, royalty-free basis to BMJ Publishing Group Ltd ("BMJ") its licensees and where the relevant Journal is co-owned by BMJ to the co-owners of the Journal, to publish the Work in this journal and any other BMJ products and to exploit all rights, as set out in our licence.

The Submitting Author accepts and understands that any supply made under these terms is made by BMJ to the Submitting Author unless you are acting as an employee on behalf of your employer or a postgraduate student of an affiliated institution which is paying any applicable article publishing charge ("APC") for Open Access articles. Where the Submitting Author wishes to make the Work available on an Open Access basis (and intends to pay the relevant APC), the terms of reuse of such Open Access shall be governed by a Creative Commons licence – details of these licences and which Creative Commons licence will apply to this Work are set out in our licence referred to above.

Other than as permitted in any relevant BMJ Author's Self Archiving Policies, I confirm this Work has not been accepted for publication elsewhere, is not being considered for publication elsewhere and does not duplicate material already published. I confirm all authors consent to publication of this Work and authorise the granting of this licence.

For peer review only

Household Medication Safety during the Covid-19 Pandemic: A Descriptive Qualitative Study Protocol

Title page

The name, postal address, e-mail, telephone, and fax numbers of the corresponding author.

Prof. Bryony Dean Franklin

Centre for Medication Safety and Service Quality, Imperial College Healthcare NHS Trust, London, UK and University College London School of Pharmacy, London, UK.

Bryony.deanfranklin@ucl.ac.uk | Tel +44 (0)20 7753 5800 | Fax - None

Author and Co-author's name	The full names, institutions, city, and country of all co-authors.
Grimes, Tamasine.	School of Pharmacy and Pharmaceutical Sciences, Trinity College Dublin, Dublin, Ireland.
Garfield, Sara.	Centre for Medication Safety and Service Quality, Imperial College Healthcare NHS Trust, London, UK and University College London School of Pharmacy, London, UK.
Kelly, Dervla.	School of Medicine, University of Limerick, Ireland and ULCaN, Health Research Institute, University of Limerick, Limerick, Ireland.
Cahill, Joan.	Centre for Innovative Human Studies, Trinity College Dublin, Dublin, Ireland.
Cromie, Sam.	Centre for Innovative Human Studies, Trinity College Dublin, Dublin, Ireland.
Wheeler, Carly.	Centre for Medication Safety and Service Quality, Imperial College Healthcare NHS Trust, London, UK.
Franklin, Bryony Dean.	Centre for Medication Safety and Service Quality, Imperial College Healthcare NHS Trust, London, UK and University College London School of Pharmacy, London, UK.

Up to five keywords or phrases suitable for use in an index (it is recommended to use MeSH terms).

MeSH: Coronavirus, pandemics, patient safety, quarantine, self-management

Word count - excluding title page, references, figures and tables.

3388

For peer review only - http://bmjopen.bmj.com/site/about/guidelines.xhtml

Household Medication Safety during the Covid-19 Pandemic: A Descriptive Qualitative Study Protocol

Abstract

Introduction

Those who are staying at home and reducing contact with other people during the Covid-19 pandemic are likely to be at greater risk of medication-related problems than the general population. This study aims to explore household medication practices by and for this population, identify practices that benefit or jeopardise medication safety, and develop a patient-centred framework of household medication practices during a pandemic, grounded in individual experiences.

Methods and analysis

This is a descriptive qualitative study using semi-structured interviews, by telephone or video call. People who are self-isolating and staying at home as much as possible and/or aged 70 years or over, and using at least one long-term medication, or their caregivers, will be eligible for inclusion. We will recruit 100 patient/ carer participants: 50 from the UK and 50 from the Republic of Ireland. Recruitment will be supported by our patient and public involvement (PPI) partners, personal networks, and social media. Individual participant consent will be sought, and interviews audio/video recorded and/or detailed notes made. A constructivist interpretivist approach to data analysis will involve use of the constant comparative method to organise the data, along with inductive analysis. Concepts based on a Safety I/II perspective will serve as sensitising concepts to support analysis and iterative development of a person-centred framework of household medication safety during a pandemic.

Ethical considerations and dissemination

This study has Trinity College Dublin, University of Limerick, and University College London ethics approvals. We plan to disseminate our findings via presentations at relevant patient/public, professional, academic and scientific meetings, and for publication in peer-reviewed journals. We will create a list of helpful strategies that participants have reported and share with participants, PPI partners and on social media.

For peer review only

Strengths and limitations of this study

This study will provide novel insights into the household medication practices of those who are staying at home and self-isolating for a long period of time during a pandemic, leading to a person-centred framework of safe household medication practices during a pandemic.

Data collection methods (telephone and video call) will enable us to reach vulnerable people who are staying at home during the pandemic, while maintaining public health guidance for avoiding face-to-face contact.

Because interviews are limited to the English language, the perspectives of people who do not speak English confidently are likely to be under-represented. In addition, the perspectives of those without telephone or internet access will not be obtained.

It is possible that theoretical saturation may not be reached due to resource constraints, which limit the capacity for recruitment to fifty participants per country.

For peer review only - http://bmjopen.bmj.com/site/about/guidelines.xhtml

Introduction

Problem statement and knowledge gap

Medication use is the most common intervention in healthcare. In normal times, more than 8% of all adult emergency hospital admissions and 25% of those for older adults (65 years and over) are related to medication use, with increasing age and higher disease burden being key risk factors (1, 2). In 2020, the world's population is predicted to use 4.5 trillion medication doses, up 24% from 2015, and more than 50% will consume more than one dose per day (3). A recent American study identified the annual cost of medication-related morbidity and mortality, consequent to non-optimised medication use, as €528 billion, equivalent to 16% of healthcare expenditure (4). As a result of such problems, the current World Health Organization (WHO) Global Patient Safety Challenge seeks to reduce the burden of serious, avoidable medication-related harm by 50% within five years from 2017 (5).

Optimal medication use requires input from multiple stakeholders, including consumers, patients and carers, operating within complex adaptive systems (6). Evidence suggests lay involvement as a potentially important modifiable factor in optimising medication outcomes and providing system resilience (7-10). Patients and family members routinely take on medication management roles (9, 11-13), often of complex medication regimens and in challenging contexts, such as in dementia (14-16), heart failure (17), chronic obstructive pulmonary disease (18), cancer (19) and home palliative care (20). However, there are known hazards in lay medication practices, including errors in medication administration (9), sharing or borrowing prescribed medication (21, 22), unsafe or unsuitable storage (23, 24) or disposal (19), hoarding (17, 25), stockpiling (17), and non-adherence (26). Therefore, there is much to address before the full potential of lay involvement in safe household medication use can be realised.

The literature suggests that medication use poses a significant body of "work" for lay users (11, 17, 18, 27). Specifically, this suggests that patients themselves manage the majority of their medication work, but that a proportion is managed by others who are members of the person's social network (11). Explorations of the nature of lay work involved in medication practices, whether for self or others, have identified a range of strands to this work. The work includes planning and coordination of medication taking and adherence, emplacement of medication in the home or other personal spaces, tracking of medication supplies, monitoring of effects of medication use, monitoring for and management of one's own or others' errors, management of information from professional or non-professional and personal sources, and interactions between all of these (11, 17, 18, 27-29). Several studies identify that lay medication work involves an emotional burden, often associated with a response to the need to use or rely on medication, the toll of the work involved and the response to one's own or another's efforts around medication adherence (11, 17, 18, 27, 28). Medication use has been identified as a tool to facilitate a person to (re)gain control in the personal context of a relatively less controlled or chaotic experience of illness (11, 17). There is evidence that medication taking, and medication work, is a highly personalised and complex phenomenon that may be contingent upon, and contextualised by, one's situation, environment, processes and network (11, 17, 30). Studies have identified that medication work comprises sociotechnical efforts that are often complex (17), and that lay people develop, adapt and rely upon a variety of strategies to achieve personal goals associated with medication use (11, 17, 18, 29, 30).

In their study of older adults with heart failure, Mickelson et al argue that medication management is the most common performed self-care behaviour in this cohort (17). They discuss the concept of

For peer review only - http://bmjopen.bmj.com/site/about/guidelines.xhtml

"medication performance", in a systems science sense, connecting the behaviours around medication practices with the achievement of outcomes or goals. Cheraghi-Sohi et al (11) developed a framework of "medication work" using social network analysis to map the work undertaken by people with long-term conditions (LTCs) and their wider social networks, i.e. people identified in some way to be of relevance to the person's medication management (11). Schafheutle et al subsequently applied and adapted this framework to people experiencing chronic obstructive pulmonary disease (18). Both Cheraghi-Sohi and Schafheutle et al's studies were based on interviews undertaken with people with LTCs and therefore provide the patient's/carer's perspective, which is also central to the present work (11, 18). However, this body of evidence has been generated during normal times, outside of the context and constraints of a pandemic, and studies have not examined populations staying at home for long periods.

During the Covid-19 pandemic, people who are self-isolating and housebound for long periods of time, whether due to age or "extreme medical vulnerability", are potentially at increased risk of medication-related problems or harm. These may include medication errors, adverse drug reactions, adverse drug events and medication non-adherence. The key clinical issues with the potential to affect medication safety for those who are self-isolating for long periods of time during the Covid-19 pandemic are: 1) disrupted routine healthcare services and supply chains; 2) altered household mobility, wellbeing and support structures; 3) reluctance to attend healthcare and 4) misinformation about medications. These create additional challenges for standard "medication performance" and medication safety, at a time when prevention of avoidable iatrogenic harm is particularly important.

The present need for research

Relatively little is known about medication safety or changes to household medication practices during pandemics or other events of major public health concern. Limited evidence on medication use during previous pandemics identifies reduced health-seeking behaviour from clinics or hospitals, increased self-management of common or chronic ailments at home, increased self-medication, stockpiling antiviral medication and increased attendance at emergency departments for anti-viral related adverse effects (31-34). Disruption of household mobility, wellbeing, and social network membership is likely for people who are staying at home and self-isolating during the Covid-19 pandemic. This disruption therefore has the potential to affect medication work, practices, and performance and to alter routine defences or hazards, thereby affecting medication safety.

Previous studies describe drug-related problems and their classification systems, typically undertaken from the healthcare professional's perspective, rather than that of the person taking the medication or their social network, and in the context of medication review, rather than the routine household settings (35). The patient's perspective of household medication use is central to the present study. The patient-centred medication safety framework (P-MEDS) is grounded in the patient's perspective of matters that they consider important to the management of medication safety incidents in primary care (8). However, it does not address the broader concept of household medication practices, which is the focus of the current study.

Evidence supports the combined use of Safety-I and Safety-II approaches to identify when, where, how and why things go wrong (Safety-I) or right (Safety-II) (36). Medication practices occur within complex sociotechnical systems where people (agents and actors), artefacts, information and knowledge flows, technologies, environments and individual or collective behaviours meet in a dynamic and unpredictable way (6, 17, 37, 38). A safety I/II perspective has previously been used to understand and create frameworks of hazards and defences associated with routine medication practices in the community (8), hospital (38) and domiciliary settings (12, 39), involving medication

For peer review only - http://bmjopen.bmj.com/site/about/guidelines.xhtml

safety incidents, intravenous infusion practices, informal carer medication administration and domiciliary medication safety among caregivers of chronically ill children. Mickelson et al used a systems engineering framework to support their analysis of medication management strategies by older patients with heart failure (17). We are not aware of any study that has applied safety I/II approaches to explore household medication practices among either the general adult population or those who are housebound, or in the context of a pandemic.

This study therefore seeks to explore household medication practices for people who are staying at home and reducing contact with others as much as possible during the Covid-19 pandemic. It also aims to develop a lay-centred framework of medication practices, grounded in individual experiences, that can be used to enable patient and carer agency in safe medication practices during the Covid-19 pandemic and beyond.

Specific objectives are to:

- Explore experiences of patients (or their carers) around household medication practices during the pandemic.
- Identify practices and situations that may benefit or jeopardise medication safety during the pandemic.
- Create a lay-centred medication practice safety framework relating to those who are self-isolating and housebound during the pandemic.

Methods and analysis

Researcher characteristics and reflexivity

The research team is composed of research pharmacists working in the academic setting (TG, DK), and hospital and academic settings (BDF, SG), an organisational psychologist (SC) and senior researchers with expertise in human factors, wellbeing management and older adult enablement (JC) and health services research (CW). Patient and public involvement partners will also be involved in data analysis.

Context

We will conduct the study in the four devolved nations of the UK, and the Republic of Ireland. All have implemented public health guidance to protect those who are extremely medically vulnerable during the Covid-19 pandemic. This includes advice for such people to stay at home as much as possible and reduce contact with other people, referred to as "cocooning" in Ireland and "shielding" in the UK.

Design

Qualitative exploration using semi-structured interviews. Qualitative methods are central to exploring medication practices and medication safety, and have been used for this purpose in both domiciliary and hospital settings (8, 12, 38, 39).

Definition of household medication practices

We define household medication practices as any activities by patients, their caregivers or members of their social networks related to obtaining supplies, deciding whether or not to use medicines,

For peer review only - http://bmjopen.bmj.com/site/about/guidelines.xhtml

storing, consuming or administering medication, monitoring, and responding to signs of therapeutic or adverse effects. We include prescribed, over-the-counter, complementary and alternative medication.

Participants

Inclusion criteria

Adults will be eligible to participate if:

- They experience a medical vulnerability listed on the government public health advice as requiring protection against Covid-19 infection and have been advised to shield or cocoon during the Covid-19 pandemic, or are aged 70 years or more; and
- They use at least one long-term medication.
  Or
- They assist in the medication management of an adult who fulfils the above criteria.

Exclusion criteria

- Children under 18 years of age,
- People too unwell or otherwise unable to consent to interview,
- People not speaking English confidently,
- People without access to a telephone or a device with internet connection.

Sampling strategy

Up to 100 adult research participants will be selected using convenience sampling, to include patients and carers living in England or the Republic of Ireland. Our protocol does not include a screening procedure to assess the demographic or clinical characteristics of prospective research participants prior to study recruitment. However, following recruitment, we will create a matrix of brief background demographic characteristics of those previously interviewed to support iterative, focussed recruitment of a diverse sample. We aim for the study population to represent people with a range of ages, genders, ethnicities, geographical areas, number of medicines and living alone or with others and those who are patients or carers.

Recruitment

Research participants will be recruited through patient or carer advocacy groups, by engaging with our public and patient involvement (PPI) partners, personal networks and through social media. Our focussed recruitment will also support us to recruit a diverse sample of people with differing at-home medication needs and roles. For example, we will do focused recruitment of those in carer roles, by engaging with our PPI partners in carer advocacy groups. We will do focussed recruitment of those experiencing particular medical vulnerabilities by engaging with charities and PPI partners working in that context, for example, cystic fibrosis, arthritis, cancer, heart and lung transplant, old age.

Data collection

We will use semi-structured interviews conducted by telephone or video call. Data will be collected from June through September 2020. Patients will be invited to express their preferred mode of data collection. We aim to interview 50 participants in Ireland and 50 in the UK. With the participant's

For peer review only - http://bmjopen.bmj.com/site/about/guidelines.xhtml

consent, interviews will be audio or audio-video recorded and/or the researcher will take detailed notes.

Interview

A member of the research team will conduct the interview at a pre-agreed date and time. The interview will explore medication use by the patient participant or for the person(s) to whom a carer participant provides care. This interview will occur remotely using the participant's preferred method of telephone or video call. At no stage will the researcher and participant meet in person. Interviews are expected to last 15-45 minutes.

The proposed topic guide (Box 1) was developed using *a priori* principles of routine lay medication use, applied in the context of the Covid-19 pandemic. Interviewers will use additional prompts and follow-up questions as needed.

Box 1, Topic guide

1. Before the coronavirus pandemic, how did you get on managing your medicines, or the medicines for the person(s) to whom you provide care?
2. Since the coronavirus pandemic started, how has the way you manage or use medicines changed?
3. Have you experienced any difficulty using or managing medication during the pandemic?
4. What do you think would make it easier or better to manage medications at home during the pandemic?
5. What would you advise others to do in your situation?
6. Do you have anything else you would like to add?
7. Background and demographic information.

Data processing

Interview recordings will be transcribed verbatim (either automated or using a third-party service) and merged with the researcher notes. Any identifiable information will be redacted from the transcription. The recording will be permanently deleted at a time consistent with the ethics approval and data protection regulations in each country. The transcribed files will be imported into NVivo to support data analysis. We will restrict access to the identifiable interview files to the research team at each site (Dublin, Limerick or London). Following irreversible anonymisation, the data collected by the researcher(s) at each university will be processed and analysed at that university and may be transferred among them at the later stages of analysis to support comparison and framework development in line with ethical approvals.

Analysis

A constructivist-interpretive analysis approach will be used. The constant comparative technique will be employed to systematically organise, compare and understand similarities and differences in the data (40). Data analysis will occur in parallel to data collection. Inductive analysis will be used to identify the emergent medication practices, strategies, hazards and defences. Sociotechnical assessment approaches using a Safety I/II perspective (36) and Schafheutle et al's medication work framework (18) will serve as sensitising concepts (41) to support the iterative development of our person-centred medication practice safety framework, relating to those who are staying at home for long periods during a pandemic.

For peer review only - http://bmjopen.bmj.com/site/about/guidelines.xhtml

Research rigour, trustworthiness and credibility

We used the Standards for Reporting Qualitative Research recommendations to guide this protocol development (42). In Ireland, research participants will be offered access to the recorded interview file, researcher notes or transcription. Member checks of these files are welcome but are not required. Each interviewer will quality assure their own interviews for transcription accuracy. A proportion of interviews will be quality assured by another member of the research team validation of coding/analysis. Publication of this protocol is our first step in documenting and creating an audit trail of decisions made throughout the project and we will continue to capture these during data collection, analysis and reporting.

Patient and public involvement

PPI will be facilitated through personal and professional networks, contacts within patient advocacy groups and professional organisations. PPI has commenced by partners validating our research aim and reviewing all participant-facing materials including recruitment materials, participant information leaflet, consent form and interview topic guide. Research participant recruitment will be supported by our PPI partners in patient and carer advocacy groups and other networks. PPI will extend to consultation on dissemination and outreach activities and involvement with data analysis, using models employed previously (43, 44). All PPI will occur remotely, compliant with current public health advice for avoiding face-to-face contact.

Ethical considerations

Approval for this study was granted by the Trinity College Dublin Faculty of Health Sciences Research Ethics Committee (reference Covid-19 2020502), University of Limerick Education and Health Sciences Research Ethics Committee (reference 2020_06_08_EHS) and University College London Research Ethics Committee (reference 18417.001). The legal basis, in Ireland and the UK, for the processing of this data is scientific research in the public interest, however, we will invite informed consent from all participants prior to participation. In the context of the Covid-19 pandemic and infection control of this medically vulnerable group, verbal consent by audio-recorded telephone or video call, in cases where hard copy postage or electronic transmission poses a challenge, has been deemed acceptable by the ethics committees concerned. Compliant with contemporary data protection guidance for qualitative research during Covid-19, recordings of interviews will be permanently deleted as soon as specified in the ethics application at each site and transcripts will be irreversibly anonymised. As with all qualitative research, quotations from interviews will be used to illustrate the findings and, in all cases, will be anonymised and of a nature that cannot be traced back to any given person. If interviewers identify any pressing medication safety concerns, they will signpost the participant to relevant sources of support.

Dissemination

We plan to present our work at suitable Irish, UK and international conferences and to publish at least one peer-reviewed research paper. We will produce plain English summaries of our work, which we will offer to send to participants, and we will engage with our PPI partners to optimise public outreach and research impact to the wider public. We will disseminate to healthcare professionals through professional bodies. We will produce a policy brief outlining key recommendations to support safer lay medication practices among those who are housebound in the context of a pandemic. Our research paper(s) will be made available open access and the

For peer review only - http://bmjopen.bmj.com/site/about/guidelines.xhtml

summaries will be shared through our university repositories and social media channels, and through our PPI partners' social media channels.

Impact

The proposed output from this research is a person-centred medication practice safety framework, relating to those who are staying at home and reducing contact with others as much as possible during the Covid-19 pandemic. We will create a list of helpful strategies that participants have reported and share this with participants, PPI partners and on social media, to use as a tool to enable agency and medication safety at home during a pandemic.

The research priorities outlined in the WHO and Global Research Collaboration for Infectious Disease Preparedness joint research roadmap for the Covid-19 pandemic include: to rapidly identify secondary effects of the outbreak and deliver strategies to mitigate patient harm, and to rapidly involve communities in the design, delivery and dissemination of research (45). The proposed study addresses both priorities by providing a person-centred framework, based on individual experiences, with significant PPI input. This framework will later be developed to enable patient and carer agency in safe medication practice during the Covid-19 pandemic and beyond. Enabling patients, carers and social network members to become agents in medication safety aligns with the WHO agreed global priorities for medication safety research (46). Future work will explore the transferability of this framework to a wider population of housebound and ambulatory people using medication long-term and in normal times outside of the context of a global pandemic. Given the forecasted increase in the global population of adults living well with age-related conditions over the coming decades (47), an important consideration is to enable people to remain in their homes and be agents in their own medication management and safety (48, 49).

Authors' contributions

TG, DK, BDF and SG led on study design. TG led on manuscript drafting. All authors contributed to manuscript revision and approval.

Funding statement

This work was supported by the Health Research Institute, University of Limerick (HRIUL) (Grant number not applicable), and the National Institute for Health Research (NIHR) Imperial Patient Safety Translational Research Centre (Grant number SF003). This report presents independent research. The views expressed in this publication are those of the authors, not necessarily those of the HRIUL, the NHS, the NIHR or the Department of Health and Social Care.

Acknowledgements

We are grateful for input from our PPI partners, including those who reviewed participant-facing materials: Alex Taylor; Marney Williams; Fran Husson; Jill Lloyd; John Norton; Mike Etkind; Dr Nikki Dunne, Research Officer, Family Carers Ireland; Corona Joyce, Senior Policy Officer, Age Action Ireland; Josephine Fogarty, Co-ordinator of Traveller Health, Midwest Community Healthcare, Health Service Executive, Ireland.

Competing interests statement.

None declared.

For peer review only - http://bmjopen.bmj.com/site/about/guidelines.xhtml

References

1. Ahern F, Sahm LJ, Lynch D, et al. Determining the frequency and preventability of adverse drug reaction-related admissions to an Irish University Hospital: a cross-sectional study. Emerg Med J. 2014; 31: 24-29.
2. Hamilton H, Gallagher P, Ryan C, et al. Potentially inappropriate medications defined by STOPP criteria and the risk of adverse drug events in older hospitalized patients. Archives of internal medicine. 2011; 171: 1013-19.
3. Aitken M, Kleinrock M. Global medicines use in 2020: outlook and implications. Parsippany, NJ: IMS Institute for Healthcare Informatics, 2015.
4. Watanabe JH, McInnis T, Hirsch JD. Cost of Prescription Drug–Related Morbidity and Mortality. Annals of Pharmacotherapy. 2018; 52: 829-37.
5. Donaldson LJ, Kelley ET, Dhingra-Kumar N, et al. Medication without harm: WHO's third global patient safety challenge. The Lancet. 2017; 389: 1680-81.
6. Werner NE, Malkana S, Gurses AP, et al. Toward a process-level view of distributed healthcare tasks: Medication management as a case study. Applied Ergonomics. 2017; 65: 255-68.
7. Fylan B, Armitage G, Naylor D, et al. A qualitative study of patient involvement in medicines management after hospital discharge: an under-recognised source of systems resilience. BMJ Quality & Safety. 2018; 27: 539-46.
8. Giles SJ, Lewis PJ, Phipps DL, et al. Capturing Patients' Perspectives on Medication Safety: The Development of a Patient-Centered Medication Safety Framework. J Patient Saf. 2019.
9. Parand A, Garfield S, Vincent C, et al. Carers' medication administration errors in the domiciliary setting: A systematic review. PloS one. 2016; 11: e0167204.
10. O'Hara JK, Aase K, Waring J. Scaffolding our systems? Patients and families 'reaching in'as a source of healthcare resilience. BMJ Quality & Safety. 2019; 28:3-6.
11. Cheraghi-Sohi S, Jeffries M, Stevenson F, et al. The influence of personal communities on the self-management of medication taking: A wider exploration of medicine work. Chronic illness. 2015; 11: 77-92.
12. Chew C-C, HSS A-S, Chan H-K, et al. Medication Safety at Home: A Qualitative Study on Caregivers of Chronically Ill Children in Malaysia. Hospital Pharmacy. 2019: DOI:10.1177/0018578719851719.
13. Sorensen L, Stokes J, Purdie D, et al. Medication management at home: medication risk factor prevalence and inter-relationships. Journal of clinical pharmacy and therapeutics. 2006; 31: 485-91.
14. Gillespie R, Mullan J, Harrison L. Managing medications: the role of informal caregivers of older adults and people living with dementia. A review of the literature. J Clin Nurs. 2014; 23: 3296-308.
15. Maidment I, Booth A, Mullan J, et al. Developing a framework for a novel multi-disciplinary, multi-agency intervention (s), to improve medication management in community-dwelling older people on complex medication regimens (MEMORABLE)—a realist synthesis. Systematic reviews. 2017; 6: 125.
16. Poland F, Mapes S, Pinnock H, et al. Perspectives of carers on medication management in dementia: lessons from collaboratively developing a research proposal. BMC Res Notes. 2014; 7: 463.
17. Mickelson RS, Holden RJ. Medication management strategies used by older adults with heart failure: a systems-based analysis. Eur J Cardiovascular Nurs. 2018; 17: 418-28.
18. Schafheutle EI, Fegan T, Ashcroft DM. Exploring medicines management by COPD patients and their social networks after hospital discharge. Int J Clin Pharm. 2018; 40: 1019-29.
19. Silvestre J, Reddy A, De La Cruz M, et al. Frequency of unsafe storage, use, and disposal practices of opioids among cancer patients presenting to the emergency department. Palliative & supportive care. 2017; 15: 638-43.

For peer review only - http://bmjopen.bmj.com/site/about/guidelines.xhtml

20. Wilson E, Caswell G, Turner N, et al. Managing Medicines for Patients Dying at Home: A Review of Family Caregivers' Experiences. Journal of Pain and Symptom Management. 2018; 56: 962-74.
21. Renny MH, Thaker RH, Dayan PS. Frequency of and Factors Associated With Prescription Medication Sharing Within Families. Pediatric Emergency Care. 2019. doi: 10.1097/PEC.0000000000001804
22. Markotic F, Jurisic D, Curkovic M, et al. Sharing of prescription analgesics amongst patients in family practice: Frequency and associated factors. Eur J Pain. 2018; 22: 716-27.
23. Kusturica MP, Sabo A, Tomic Z, et al. Storage and disposal of unused medications: knowledge, behavior, and attitudes among Serbian people. Int J Clin Pharm. pharmacy. 2012; 34: 604-10.
24. Hewson C, Shen CC, Strachan C, et al. Personal medicines storage in New Zealand. J Primary Health Care. 2013; 5: 146-50.
25. VanDyke MM, Steffen AM. Medication saving behaviors of older adults: Scale developed to assess family caregiver perspectives. Clin Gerontologist. 2017; 40: 258-67.
26. Brown MT, Bussell JK. Medication adherence: WHO cares? Mayo Clin Proc. 2011; 86: 304-14.
27. Pound P, Britten N, Morgan M, et al. Resisting medicines: a synthesis of qualitative studies of medicine taking. Soc Sci & Med. 2005; 61: 133-55.
28. McCoy L. Time, self and the medication day: a closer look at the everyday work of ‘adherence’. Sociology of health & illness. 2009; 31: 128-46.
29. Townsend A, Hunt K, Wyke S. Managing multiple morbidity in mid-life: a qualitative study of attitudes to drug use. BMJ. 2003; 327: 837.
30. Dew K, Chamberlain K, Hodgetts D, et al. Home as a hybrid centre of medication practice. Sociology of Health & Illness. 2014; 36: 28-43.
31. Bali S, Stewart KA, Pate MA. Long shadow of fear in an epidemic: fearonomic effects of Ebola on the private sector in Nigeria. BMJ Global Health. 2016; 1: e000111.
32. Gasink LB, Linkin DR, Fishman NO, et al. Stockpiling drugs for an avian influenza outbreak: examining the surge in oseltamivir prescriptions during heightened media coverage of the potential for a worldwide pandemic. Infection Control & Hospital Epidemiology. 2009; 30: 370-76.
33. Lovegrove MC, Shehab N, Hales CM, et al. Emergency department visits for antiviral adverse events during the 2009 H1N1 influenza pandemic. Public Health Reports. 2011; 126: 312-17.
34. McLean KE, Abramowitz SA, Ball JD, et al. Community-based reports of morbidity, mortality, and health-seeking behaviours in four Monrovia communities during the West African Ebola epidemic. Global Public Health. 2018; 13: 528-44.
35. Basger BJ, Moles RJ, Chen TF. Application of drug-related problem (DRP) classification systems: a review of the literature. Eur J Clin Pharm. 2014; 70: 799-815.
36. Carson-Stevens A, Donaldson L, Sheikh A. The Rise of Patient Safety-II: Should We Give Up Hope on Safety-I and Extracting Value From Patient Safety Incidents?: Comment on" False Dawns and New Horizons in Patient Safety Research and Practice". Int J Health Policy & Management. 2018; 7: 667.
37. Abebe E, Scanlon MC, Lee KJ, et al. What do family caregivers do when managing medications for their children with medical complexity? Applied Ergonomics. 2020; 87: 103108.
38. Furniss D, Mayer A, Franklin BD, et al. Exploring structure, agency and performance variability in everyday safety: An ethnographic study of practices around infusion devices using distributed cognition. Safety Science. 2019; 118: 687-701.
39. Parand A, Faiella G, Franklin BD, et al. A prospective risk assessment of informal carers’ medication administration errors within the domiciliary setting. Ergonomics. 2018; 61: 104-21.
40. Pope C, Mays N. Synthesising qualitative research. Qualitative Research in Health care. 2006; 142: 152.
41. Bowen GA. Grounded theory and sensitizing concepts. Int J Qual Methods. 2006; 5: 12-23.

For peer review only - http://bmjopen.bmj.com/site/about/guidelines.xhtml

42. O'Brien BC, Harris IB, Beckman TJ, et al. Standards for reporting qualitative research: a synthesis of recommendations. Academic Medicine. 2014; 89: 1245-51.
43. Furniss D, Iacovides I, Lyons I, et al. Patient and public involvement in patient safety research: a workshop to review patient information, minimise psychological risk and inform research. Research Involvement and Engagement. 2016; 2: 19.
44. Garfield S, Jheeta S, Husson F, et al. Lay involvement in the analysis of qualitative data in health services research: a descriptive study. Research Involvement and Engagement. 2016; 2: 29.
45. Organization WH, Preparedness GRCfID. 2019 Novel Coronavirus Global Research and Innovation Forum: Towards a Research Roadmap Geneva, Switzerland: World Health Organization, 2020.
46. Sheikh A, Rudan I, Cresswell K, et al. Agreeing on global research priorities for medication safety: an international prioritisation exercise. J Global Health. 2019; 9.
47. Chang AY, Skirbekk VF, Tyrovolas S, et al. Measuring population ageing: an analysis of the Global Burden of Disease Study 2017. The Lancet Public Health. 2019; 4: e159-e67.
48. Slaintecare Report. Houses of the Oireachtas Committee, 2017. Dublin, Ireland. https://www.gov.ie/en/publication/0d2d60-slaintecare-publications/#the-slaintecare-report Accessed 2020-07-14.
49. National Institute for Health and Care Excellence. NICE Guidelines 67 Managing medicines for adults receiving social care in the community., 2017. https://www.nice.org.uk/guidance/ng67 Accessed 2020-07-14.

For peer review only

For peer review only - http://bmjopen.bmj.com/site/about/guidelines.xhtml

SRQR Checklist

Item	Comments for protocol	Comments for full manuscript
Item 1. Title: Concise description of the nature and topic of the study. Identifying the study as qualitative or indicating the approach (e.g., ethnography, grounded theory) or data collection methods (e.g., interview, focus group) is recommended.	Done, title, page 1	Same
Item 2. Abstract: Summary of key elements of the study using the abstract format of the intended publication; typically includes background, purpose, methods, results, and conclusions.	Done, abstract, page 1	
Item 3. Problem Formulation: Description and significance of the problem/phenomenon studied; review of relevant theory and empirical work; problem statement.	Problem statement, current need for this research, pages 3-4	
Item 4. Purpose or research question: Purpose of the study and specific objectives or questions.	Current need for this research, pages 3-4	
Item 5. Qualitative approach and research paradigm: Qualitative approach (e.g., ethnography, grounded theory, case study, phenomenology, narrative research) and guiding theory if appropriate; identifying the research paradigm (e.g., post-positivist, constructivist/interpretivist) is also recommended; rationale.	Described in methods, pages 4-5	
Item 6. Researcher characteristics and reflexivity: Researchers' characteristics that may influence the research, including personal attributes, qualifications/experience, relationship with participants, assumptions, and/or presuppositions; potential or actual interaction between researchers' characteristics and the research questions, approach, methods, results and/or transferability.	Described in methods, page 4	

For peer review only

Item 7. Context: Setting/site and salient contextual factors; rationale.	Methods, pages 4-5	
Item 8. Sampling strategy: How and why research participants, documents, or events were selected; criteria for deciding when no further sampling was necessary (e.g., sampling saturation); rationale.	Methods, page 5	
Item 9. Ethical issues pertaining to human subjects: Documentation of approval by an appropriate ethics review board and participant consent, or explanation for lack thereof; other confidentiality and data security issues.	Methods, page 7	
Item 10. Data collection methods: Types of data collected; details of data collection procedures including (as appropriate) start and stop dates of data collection and analysis, iterative process, triangulation of sources/methods, and modification of procedures in response to evolving study findings; rationale.	Methods, pages 5-7	
Item 11. Data collection instruments and technologies: Description of instruments (e.g., interview guides, questionnaires) and devices (e.g., audio recorders) used for data collection; if/how the instrument(s) changed over the course of the study.	Methods, page 6	
Item 12. Units of study: Number and relevant characteristics of participants, documents, or events included in the study; level of participation.	Methods, page 6-7	
Item 13. Data processing: Methods for processing data prior to and during analysis, including transcription, data entry, data management and security, verification of data integrity, data coding and anonymization / de-identification of excerpts.	Methods, page 6	
Item 14. Data analysis: Process by which inferences, themes, etc. were identified and developed, including	Methods, pages 6-7	

For peer review only

the researchers involved in data analysis; usually references a specific paradigm or approach; rationale.		
Item 15. Techniques to enhance trustworthiness: Techniques to enhance trustworthiness and credibility of data analysis, (e.g., member checking, triangulation, audit trail); rationale.	Methods, page 7	
Item 16. Synthesis and interpretation: Main findings (e.g., interpretations, inferences, and themes); might include development of a theory or model, or integration with prior research or theory.	Methods, within analysis, pages 6-7	
Item 17. Links to empirical data: Evidence (e.g., quotes, field notes, text excerpts, photographs) to substantiate analytic findings.	N/A at protocol stage	
Item 18. Integration with prior work, implications, transferability, and contribution(s) to the field: Short summary of main findings, explanation of how findings and conclusions connect to, support, elaborate on, or challenge conclusions of earlier scholarship; discussion of scope of application/generalizability; identification of unique contribution(s) to scholarship in a discipline or field.	N/A at protocol stage	
Item 19. Limitations: Trustworthiness and limitations of findings.	N/A at protocol stage	
Item 20. Conflicts of interest: Potential sources of influence or perceived influence on study conduct and conclusions; how these were managed.	Page 9	
Item 21. Funding: Sources of funding and other support; role of funders in data collection, interpretation, and reporting.	Page 8	

For peer review only

Household Medication Safety Practices during the Covid-19 Pandemic: A Descriptive Qualitative Study Protocol

Journal:	*BMJ Open*
Manuscript ID	bmjopen-2020-044441.R1
Article Type:	Protocol
Date Submitted by the Author:	27-Oct-2020
Complete List of Authors:	Grimes, Tamasine C.; Trinity College, School of Pharmacy<br>Garfield, Sara; University College London, School of Pharmacy<br>Kelly, Dervla ; University of Limerick, Graduate Entry Medical School<br>Cahill, Joan; Trinity College Dublin, Centre for Innovative Human Studies<br>Cromie, Sam; Trinity College Dublin, Centre for Innovative Human Studies<br>Wheeler, Carly; Imperial College Healthcare NHS Trust, Centre for Medication Safety and Service Quality<br>Dean Franklin, Bryony; University College London School of Pharmacy,
**Primary Subject Heading**:	Qualitative research
Secondary Subject Heading:	Qualitative research, Public health
Keywords:	Public health < INFECTIOUS DISEASES, COVID-19, PRIMARY CARE, QUALITATIVE RESEARCH, THERAPEUTICS

SCHOLARONE™ Manuscripts

I, the Submitting Author has the right to grant and does grant on behalf of all authors of the Work (as defined in the below author licence), an exclusive licence and/or a non-exclusive licence for contributions from authors who are: i) UK Crown employees; ii) where BMJ has agreed a CC-BY licence shall apply, and/or iii) in accordance with the terms applicable for US Federal Government officers or employees acting as part of their official duties; on a worldwide, perpetual, irrevocable, royalty-free basis to BMJ Publishing Group Ltd ("BMJ") its licensees and where the relevant Journal is co-owned by BMJ to the co-owners of the Journal, to publish the Work in this journal and any other BMJ products and to exploit all rights, as set out in our licence.

The Submitting Author accepts and understands that any supply made under these terms is made by BMJ to the Submitting Author unless you are acting as an employee on behalf of your employer or a postgraduate student of an affiliated institution which is paying any applicable article publishing charge ("APC") for Open Access articles. Where the Submitting Author wishes to make the Work available on an Open Access basis (and intends to pay the relevant APC), the terms of reuse of such Open Access shall be governed by a Creative Commons licence – details of these licences and which Creative Commons licence will apply to this Work are set out in our licence referred to above.

Other than as permitted in any relevant BMJ Author's Self Archiving Policies, I confirm this Work has not been accepted for publication elsewhere, is not being considered for publication elsewhere and does not duplicate material already published. I confirm all authors consent to publication of this Work and authorise the granting of this licence.

For peer review only

Household Medication Safety Practices during the Covid-19 Pandemic: A Descriptive Qualitative Study Protocol

Title page

The name, postal address, e-mail, telephone, and fax numbers of the corresponding author.

Prof. Bryony Dean Franklin

Centre for Medication Safety and Service Quality, Imperial College Healthcare NHS Trust, London, UK and University College London School of Pharmacy, London, UK.

Bryony.deanfranklin@ucl.ac.uk | Tel +44 (0)20 7753 5800 | Fax - None

Author and Co-author's name	The full names, institutions, city, and country of all co-authors.
Grimes, Tamasine.	School of Pharmacy and Pharmaceutical Sciences, Trinity College Dublin, Dublin, Ireland.
Garfield, Sara.	Centre for Medication Safety and Service Quality, Imperial College Healthcare NHS Trust, London, UK and University College London School of Pharmacy, London, UK.
Kelly, Dervla.	School of Medicine, University of Limerick, Ireland and ULCaN, Health Research Institute, University of Limerick, Limerick, Ireland.
Cahill, Joan.	Centre for Innovative Human Studies, Trinity College Dublin, Dublin, Ireland.
Cromie, Sam.	Centre for Innovative Human Studies, Trinity College Dublin, Dublin, Ireland.
Wheeler, Carly.	Centre for Medication Safety and Service Quality, Imperial College Healthcare NHS Trust, London, UK.
Franklin, Bryony Dean.	Centre for Medication Safety and Service Quality, Imperial College Healthcare NHS Trust, London, UK and University College London School of Pharmacy, London, UK.

Up to five keywords or phrases suitable for use in an index (it is recommended to use MeSH terms).

MeSH: Coronavirus, pandemics, patient safety, quarantine, self-management

Word count - excluding title page, references, figures and tables.

3406

For peer review only - http://bmjopen.bmj.com/site/about/guidelines.xhtml

Household Medication Safety Practices during the Covid-19 Pandemic: A Descriptive Qualitative Study Protocol

Abstract

Introduction

Those who are staying at home and reducing contact with other people during the Covid-19 pandemic are likely to be at greater risk of medication-related problems than the general population. This study aims to explore household medication practices by and for this population, identify practices that benefit or jeopardise medication safety, and develop best practice guidance about household medication safety practices during a pandemic, grounded in individual experiences.

Methods and analysis

This is a descriptive qualitative study using semi-structured interviews, by telephone or video call. People who have been advised to 'cocoon'/'shield' and/or are aged 70 years or over, and using at least one long-term medication, or their caregivers, will be eligible for inclusion. We will recruit 100 patient/ carer participants: 50 from the UK and 50 from the Republic of Ireland. Recruitment will be supported by our patient and public involvement (PPI) partners, personal networks, and social media. Individual participant consent will be sought, and interviews audio/video recorded and/or detailed notes made. A constructivist interpretivist approach to data analysis will involve use of the constant comparative method to organise the data, along with inductive analysis. From this, we will iteratively develop best practice guidance about household medication safety practices during a pandemic from the patient/carer perspective.

Ethical considerations and dissemination

This study has Trinity College Dublin, University of Limerick, and University College London ethics approvals. We plan to disseminate our findings via presentations at relevant patient/public, professional, academic and scientific meetings, and for publication in peer-reviewed journals. We will create a list of helpful strategies that participants have reported and share this with participants, PPI partners and on social media.

Strengths and limitations of this study

This study will provide novel insights into the household medication safety practices of those who are cocooning/shielding during a pandemic, leading to the creation of best practice guidance about household medication safety practices during a pandemic from the patient/carer perspective.

Data collection methods (telephone and video call) will enable us to reach vulnerable people who are staying at home during the pandemic, while maintaining public health guidance for avoiding face-to-face contact.

Because interviews are limited to the English language, the perspectives of people who do not speak English confidently are likely to be under-represented. In addition, the perspectives of those without telephone or internet access will not be obtained.

It is possible that theoretical saturation may not be reached due to resource constraints, which limit the capacity for recruitment to fifty participants per country.

For peer review only - http://bmjopen.bmj.com/site/about/guidelines.xhtml

Introduction

Problem statement and knowledge gap

Medication use is the most common intervention in healthcare. In the past decade, prior to the COVID-19 pandemic , more than 8% of all adult emergency hospital admissions and 25% of those for older adults (65 years and over) have been related to medication use, with increasing age and higher disease burden being key risk factors (1, 2). In 2020, the world's population is predicted to use 4.5 trillion medication doses, up 24% from 2015, and more than 50% will consume more than one dose per day (3). A recent American study identified the annual cost of medication-related morbidity and mortality, consequent to non-optimised medication use, as €528 billion, equivalent to 16% of healthcare expenditure (4). As a result of such problems, the current World Health Organization (WHO) Global Patient Safety Challenge seeks to reduce the burden of serious, avoidable medication-related harm by 50% within five years from 2017 (5).

Optimal medication use requires input from multiple stakeholders, including consumers, patients and carers, operating within complex adaptive systems (6). Evidence suggests that contribution by patients, informal carers, family members ('lay involvement') is a potentially important modifiable factor in optimising medication outcomes and providing system resilience (7-10). Examples of lay involvement include patients and family members routinely taking on medication management roles (9, 11-13), often of complex medication regimens and in challenging contexts, such as in dementia (14-16), heart failure (17), chronic obstructive pulmonary disease (18), cancer (19) and home palliative care (20). However, there are known hazards in lay medication practices, including errors in medication administration (9), sharing or borrowing prescribed medication (21, 22), unsafe or unsuitable storage (23, 24) or disposal (19), hoarding (17, 25), stockpiling (17), and non-adherence (26). Therefore, there is much to address before the full potential of lay involvement in safe household medication use can be realised.

The literature suggests that medication use poses a significant body of medication-related "work" for lay users (11, 17, 18, 27). Specifically, this suggests that patients themselves manage the majority of their medication work, but that a proportion is managed by others who are members of the person's social network (11). Explorations of the nature of lay work involved in medication practices, whether for self or others, have identified a range of strands to this work. The work includes planning and coordination of medication taking and adherence, emplacement of medication in the home or other personal spaces, tracking of medication supplies, monitoring of effects of medication use, monitoring for and management of one's own or others' errors, management of information from professional or non-professional and personal sources, and interactions between all of these (11, 17, 18, 27-29). Several studies identify that lay medication work involves an emotional burden, often associated with a response to the need to use or rely on medication, the toll of the work involved and the response to one's own or another's efforts around medication adherence (11, 17, 18, 27, 28). Medication use has been identified as a tool to facilitate a person to (re)gain control in the personal context of a relatively less controlled or chaotic experience of illness (11, 17). There is evidence that medication taking, and medication work, are highly personalised and complex phenomena that may be contingent upon, and contextualised by, one's situation, environment, processes and network (11, 17, 30). Examples of such complexities include the medication user's individualised routines and strategies, the nature of medication(s) involved, the range of people involved, or the individual's illness burden (11). Studies have identified that medication work comprises sociotechnical efforts that are often complex (17), and

For peer review only - http://bmjopen.bmj.com/site/about/guidelines.xhtml

that lay people develop, adapt and rely upon a variety of strategies to achieve personal goals associated with medication use (11, 17, 18, 29, 30).

In their study of older adults with heart failure, Mickelson et al argue that medication management is the most common performed self-care behaviour in this cohort (17. Cheraghi-Sohi et al (11) developed a framework of “medication work” using social network analysis to map the work undertaken by people with long-term conditions (LTCs) and their wider social networks, i.e. people identified in some way to be of relevance to the person’s medication management (11). Schafheutle et al subsequently applied and adapted this framework to people experiencing chronic obstructive pulmonary disease (18). Both Cheraghi-Sohi and Schafheutle et al’s studies were based on interviews undertaken with people with LTCs and therefore provide the patient’s/carer’s perspective, which is also central to the present work (11, 18). However, this body of evidence has been generated outside of the context and constraints of a pandemic and studies have not examined populations staying at home for long periods.

During the Covid-19 pandemic, people who are self-isolating and housebound for long periods of time, whether due to age or “extreme medical vulnerability”, are potentially at increased risk of medication-related problems or harm. These may include medication errors, adverse drug reactions, adverse drug events and medication non-adherence. The key clinical issues with the potential to affect medication safety for those who are self-isolating for long periods of time during the Covid-19 pandemic are: 1) disrupted routine healthcare services and supply chains; 2) altered household mobility, wellbeing and support structures; 3) reluctance to attend healthcare or restrictions on attending healthcare and 4) misinformation about medications reported to affect the risk or severity of Covid-19 infection. These create additional challenges for standard medication safety, at a time when prevention of avoidable iatrogenic harm is particularly important.

The present need for research

Relatively little is known about medication safety or changes to household medication practices during a pandemic. Limited evidence on medication use during previous pandemics identifies reduced health-seeking behaviour from clinics or hospitals, increased self-management of common or chronic ailments at home, increased self-medication, stockpiling antiviral medication and increased attendance at emergency departments for anti-viral related adverse effects (31-34). Disruption of household mobility, wellbeing, and social network membership is likely for people who are staying at home and self-isolating during the Covid-19 pandemic. This disruption therefore has the potential to affect medication work and practices, and to alter routine defences or hazards, thereby affecting medication safety.

Previous studies have generally classified drug-related problems from a healthcare professional perspective, rather than that of the person taking the medication or their social network; drug-related problems are also generally identified in the context of medication review, rather than routine household settings (35). The patient’s perspective of household medication use is central to the present study. The patient-centred medication safety framework (P-MEDS) is grounded in the patient’s perspective of matters that they consider important to the management of medication safety incidents in primary care (8). However, it does not address the broader concept of household medication safety practices, which is the focus of the current study.

Evidence supports the combined use of Safety-I and Safety-II approaches to identify when, where, how and why things go wrong (Safety-I) or right (Safety-II) (36). Medication practices occur within complex sociotechnical systems where people (agents and actors), artefacts, information and

For peer review only - http://bmjopen.bmj.com/site/about/guidelines.xhtml

knowledge flows, technologies, environments and individual or collective behaviours meet in a dynamic and unpredictable way (6, 17, 37, 38). A safety I/II perspective has previously been used to understand and create frameworks of hazards and defences associated with routine medication practices in the community (8), hospital (38) and domiciliary settings (12, 39), involving medication safety incidents, intravenous infusion practices, informal carer medication administration and domiciliary medication safety among caregivers of chronically ill children. Mickelson et al used a systems engineering framework to support their analysis of medication management strategies by older patients with heart failure (17). We are not aware of any study that has employed safety I/II approaches to support the analysis of household medication safety practices among either the general adult population or those who are housebound, or in the context of a pandemic.

This study therefore seeks to explore household medication practices for people who are staying at home and reducing contact with others as much as possible during the Covid-19 pandemic. It also aims to create best practice guidance about household medication safety practices during a pandemic from the patient/carer perspective, that can be used to enable patient and carer agency in safe medication practices during the Covid-19 pandemic and beyond.

Specific objectives are to:

- Explore experiences of patients (or their carers) around household medication safety practices during the pandemic.
- Identify practices and situations that may benefit or jeopardise medication safety practices during the pandemic.
- Create best practice guidance about household medication safety practices during a pandemic from the patient/carer.

Methods and analysis

Researcher characteristics and reflexivity

The research team is composed of research pharmacists working in the academic setting (TG, DK), and hospital and academic settings (BDF, SG), an organisational psychologist (SC) and senior researchers with expertise in human factors, wellbeing management and older adult enablement (JC) and health services research (CW). Patient and public involvement partners will also be involved in data analysis.

Context

We will conduct the study in the four devolved nations of the UK, and the Republic of Ireland. All have implemented public health guidance to protect those who are extremely medically vulnerable during the Covid-19 pandemic. This includes advice for such people to stay at home as much as possible and reduce contact with other people, referred to as "*cocooning*" in Ireland and "*shielding*" in the UK (40, 41).

Design

Qualitative exploration using semi-structured interviews. Qualitative methods are central to exploring medication practices and medication safety, and have been used for this purpose in both domiciliary and hospital settings (8, 12, 38, 39).

For peer review only - http://bmjopen.bmj.com/site/about/guidelines.xhtml

Definition of household medication safety practices

We define household medication safety practices as any activities by patients, their caregivers or members of their social networks related to obtaining supplies, deciding whether or not to use medicines, storing, consuming or administering medication, monitoring, and responding to signs of therapeutic or adverse effects. We include prescribed, over-the-counter, complementary and alternative medication.

Participants

Inclusion criteria

Adults will be eligible to participate if they experience a medical vulnerability listed on the government public health advice as requiring protection against Covid-19 infection and have been advised to "*shield*" (40) or "*cocoon*" (41) during the Covid-19 pandemic, or are aged 70 years or more, AND they use at least one long-term medication. Carers who assist in the medication management of an adult who fulfils these two criteria are also eligible to take part.

Exclusion criteria

- Children under 18 years of age,
- People too unwell or otherwise unable to consent to interview,
- People not speaking English confidently,
- People without access to a telephone or a device with internet connection.

Sampling strategy

Up to 100 adult research participants will be selected using convenience sampling, to include patients and carers living in England or the Republic of Ireland. Our protocol does not include a screening procedure to assess the demographic or clinical characteristics of prospective research participants prior to study recruitment. However, following recruitment, we will create a matrix of brief background demographic characteristics of those previously interviewed to support iterative, focussed recruitment of a diverse sample. We aim for the study population to represent people with a range of ages, genders, ethnicities, geographical areas, number of medicines and living alone or with others and those who are patients or carers.

Recruitment

Research participants will be recruited through patient or carer advocacy groups or charities, by engaging with our public and patient involvement (PPI) partners, personal or professional networks and through social media. Our focussed recruitment will also support us to recruit a diverse sample of people with differing at-home medication needs and roles. For example, we will do focused recruitment of those in carer roles, by engaging with our PPI partners in carer advocacy groups. We will do focussed recruitment of those experiencing particular medical vulnerabilities by engaging with charities and PPI partners working in that context, for example, cystic fibrosis, arthritis, cancer, ~~heart and lung~~ organ transplant, old age. Prospective participants will be encouraged to make contact with a member of the research team, by telephone or email. Consistent with ethical, data protection and confidentiality regulations, we will not ask third parties to pass prospective participants' personal details to the research team.

For peer review only - http://bmjopen.bmj.com/site/about/guidelines.xhtml

Data collection

We will use semi-structured interviews conducted by telephone or video call. Data will be collected from June through October 2020. Patients will be invited to express their preferred mode of data collection. We aim to interview 50 participants in Ireland and 50 in the UK. With the participant's consent, interviews will be audio or audio-video recorded and/or the researcher will take detailed notes.

Interview

A member of the research team will conduct the interview at a pre-agreed date and time. The interview will explore medication use by the patient participant or for the person(s) to whom a carer participant provides care. This interview will occur remotely using the participant's preferred method of telephone or video call. At no stage will the researcher and participant meet in person. Interviews are expected to last 15-45 minutes.

The proposed topic guide (Box 1) was developed using *a priori* principles of routine lay medication use, applied in the context of the Covid-19 pandemic. Interviewers will use additional prompts and follow-up questions as needed.

Box 1, Topic guide

1. Before the coronavirus pandemic, how did you get on managing your medicines, or the medicines for the person(s) to whom you provide care?
2. Since the coronavirus pandemic started, how has the way you manage or use medicines changed?
3. Have you experienced any difficulty using or managing medication during the pandemic?
4. What do you think would make it easier or better to manage medications at home during the pandemic?
5. What would you advise others to do in your situation?
6. Do you have anything else you would like to add?
7. Background and demographic information.

Data processing

Interview recordings will be transcribed verbatim (either automated or using a third-party service) and merged with the researcher notes. Any identifiable information will be redacted from the transcription. The recording will be permanently deleted at a time consistent with the ethics approval and data protection regulations in each country. The transcribed files will be imported into NVivo to support data analysis. We will restrict access to the identifiable interview files to the research team at each site (Dublin, Limerick or London). Following irreversible anonymisation, the data collected by the researcher(s) at each university will be processed and analysed at that university and may be transferred among them at the later stages of analysis to support comparison in line with ethical approvals.

Analysis

A constructivist-interpretive analysis approach will be used. The constant comparative technique will be employed to systematically organise, compare and understand similarities and differences in the data (42). Data analysis will occur in parallel to data collection. Inductive analysis will be used to identify the emergent medication practices, strategies, hazards and defences. Sociotechnical assessment approaches using a Safety I/II perspective (36) and Schafheutle et al's medication work

For peer review only - http://bmjopen.bmj.com/site/about/guidelines.xhtml

framework (18) will serve as sensitising concepts (43) to explore the data, in which their application will be used to inspire insights appropriate to the data rather than being used as a rigid coding framework. This will support the iterative development of our best practice guidance about household medication safety practices relating to those who are staying at home for long periods during a pandemic.

Research rigour, trustworthiness and credibility

We used the Standards for Reporting Qualitative Research recommendations to guide this protocol development (44). In Ireland, research participants will be offered access to the recorded interview file, researcher notes or transcription. Member checks of these files are welcome but are not required. Each interviewer will quality assure their own interviews for transcription accuracy. A proportion of interviews will be coded independently by at least two members of the research team, followed by a comparison of and discussion of coding as a means to quality assure the coding process. Publication of this protocol is our first step in documenting and creating an audit trail of decisions made throughout the project and we will continue to capture these during data collection, analysis and reporting.

Patient and public involvement

PPI will be facilitated through personal and professional networks, contacts within patient advocacy groups, charities and professional organisations. PPI commenced during the development phase by partners, including patients, validating our research aim and reviewing all participant-facing materials including recruitment materials, participant information leaflet, consent form and interview topic guide. Research participant recruitment will be supported by our PPI partners in patient and carer advocacy groups and other networks. PPI will extend to consultation on dissemination and outreach activities and involvement with data analysis and interpretation, using models employed previously (45, 46). All PPI will occur remotely, compliant with current public health advice for avoiding face-to-face contact. Our PPI partners will be offered the opportunity to co-author the future papers describing our research findings.

Ethical considerations

Approval for this study was granted by the Trinity College Dublin Faculty of Health Sciences Research Ethics Committee (reference Covid-19 2020502), University of Limerick Education and Health Sciences Research Ethics Committee (reference 2020_06_08_EHS) and University College London Research Ethics Committee (reference 18417.001). The legal basis, in Ireland and the UK, for the processing of this data is scientific research in the public interest, however, we will invite informed consent from all participants prior to participation. In the context of the Covid-19 pandemic and infection control of this medically vulnerable group, verbal consent by audio-recorded telephone or video call, in cases where hard copy postage or electronic transmission poses a challenge, has been deemed acceptable by the ethics committees concerned. Compliant with contemporary data protection guidance for qualitative research during Covid-19, recordings of interviews will be permanently deleted as soon as specified in the ethics application at each site and transcripts will be irreversibly anonymised. As with all qualitative research, quotations from interviews will be used to illustrate the findings and, in all cases, will be anonymised and of a nature that cannot be traced back to any given person. If interviewers identify any pressing medication safety concerns, they will signpost the participant to relevant sources of support.

For peer review only - http://bmjopen.bmj.com/site/about/guidelines.xhtml

Dissemination

We plan to present our work at suitable Irish, UK and international conferences and to publish at least one peer-reviewed research paper. We will produce plain English summaries of our work, which we will offer to send to participants, and we will engage with our PPI partners to optimise public outreach and research impact to the wider public. We will disseminate to healthcare professionals through professional bodies. We will produce a policy brief outlining key recommendations to support safer lay medication practices among those who are housebound in the context of a pandemic. Our research paper(s) will be made available open access and the summaries will be shared through our university repositories and social media channels, and through our PPI partners' social media channels.

Impact

The proposed output from this research is a set of best practice guidance about household medication safety practices during a pandemic from the patient/carer perspective, relating to those who are staying at home and reducing contact with others as much as possible during the Covid-19 pandemic. We will create a list of helpful strategies that participants have reported and share this with participants, PPI partners and on social media, to use as a tool to enable agency and medication safety at home for people who are shielding, cocooning or equivalent during a pandemic.

The research priorities outlined in the WHO and Global Research Collaboration for Infectious Disease Preparedness joint research roadmap for the Covid-19 pandemic include: to rapidly identify secondary effects of the outbreak and deliver strategies to mitigate patient harm, and to rapidly involve communities in the design, delivery and dissemination of research (47). The proposed study addresses both priorities by providing person-centred guidance, based on individual experiences, with significant PPI input. Enabling patients, carers and social network members to become agents in medication safety aligns with the WHO agreed global priorities for medication safety research (48). Future work will explore the transferability of this guidance to a wider population of housebound and ambulatory people who are using medication long-term and outside of the context of a global pandemic. Given the forecasted increase in the global population of adults living well with age-related conditions over the coming decades (49), an important consideration is to enable people to remain in their homes and be agents in their own medication management and safety (50, 51).

Authors' contributions

TG, DK, BDF and SG led on study design. TG led on manuscript drafting. All (TG, DK, BDF, SG, JC, SC, CW) authors contributed to manuscript revision and approval.

Funding statement

This work was supported by the Health Research Institute, University of Limerick (HRIUL) (Grant number not applicable), and the National Institute for Health Research (NIHR) Imperial Patient Safety Translational Research Centre (Grant number SF003). This report presents independent research. The views expressed in this publication are those of the authors, not necessarily those of the HRIUL, the NHS, the NIHR or the Department of Health and Social Care.

Acknowledgements

We are grateful for input from our PPI partners, including those who reviewed participant-facing materials: Alex Taylor; Marney Williams; Fran Husson; Jill Lloyd; John Norton; Mike Etkind; Dr Nikki

For peer review only - http://bmjopen.bmj.com/site/about/guidelines.xhtml

Dunne, Research Officer, Family Carers Ireland; Corona Joyce, Senior Policy Officer, Age Action Ireland; Josephine Fogarty, Co-ordinator of Traveller Health, Midwest Community Healthcare, Health Service Executive, Ireland.

Competing interests statement.

None declared.

References

1. Ahern F, Sahm LJ, Lynch D, et al. Determining the frequency and preventability of adverse drug reaction-related admissions to an Irish University Hospital: a cross-sectional study. Emerg Med J. 2014; 31: 24-29.
2. Hamilton H, Gallagher P, Ryan C, et al. Potentially inappropriate medications defined by STOPP criteria and the risk of adverse drug events in older hospitalized patients. Archives of internal medicine. 2011; 171: 1013-19.
3. Aitken M, Kleinrock M. Global medicines use in 2020: outlook and implications. Parsippany, NJ: IMS Institute for Healthcare Informatics, 2015.
4. Watanabe JH, McInnis T, Hirsch JD. Cost of Prescription Drug–Related Morbidity and Mortality. Annals of Pharmacotherapy. 2018; 52: 829-37.
5. Donaldson LJ, Kelley ET, Dhingra-Kumar N, et al. Medication without harm: WHO's third global patient safety challenge. The Lancet. 2017; 389: 1680-81.
6. Werner NE, Malkana S, Gurses AP, et al. Toward a process-level view of distributed healthcare tasks: Medication management as a case study. Applied Ergonomics. 2017; 65: 255-68.
7. Fylan B, Armitage G, Naylor D, et al. A qualitative study of patient involvement in medicines management after hospital discharge: an under-recognised source of systems resilience. BMJ Quality & Safety. 2018; 27: 539-46.
8. Giles SJ, Lewis PJ, Phipps DL, et al. Capturing Patients' Perspectives on Medication Safety: The Development of a Patient-Centered Medication Safety Framework. J Patient Saf. 2019.
9. Parand A, Garfield S, Vincent C, et al. Carers' medication administration errors in the domiciliary setting: A systematic review. PloS one. 2016; 11: e0167204.
10. O'Hara JK, Aase K, Waring J. Scaffolding our systems? Patients and families 'reaching in'as a source of healthcare resilience. BMJ Quality & Safety. 2019; 28:3-6.
11. Cheraghi-Sohi S, Jeffries M, Stevenson F, et al. The influence of personal communities on the self-management of medication taking: A wider exploration of medicine work. Chronic illness. 2015; 11: 77-92.
12. Chew C-C, HSS A-S, Chan H-K, et al. Medication Safety at Home: A Qualitative Study on Caregivers of Chronically Ill Children in Malaysia. Hospital Pharmacy. 2019: DOI:10.1177/0018578719851719.
13. Sorensen L, Stokes J, Purdie D, et al. Medication management at home: medication risk factor prevalence and inter-relationships. Journal of clinical pharmacy and therapeutics. 2006; 31: 485-91.
14. Gillespie R, Mullan J, Harrison L. Managing medications: the role of informal caregivers of older adults and people living with dementia. A review of the literature. J Clin Nurs. 2014; 23: 3296-308.
15. Maidment I, Booth A, Mullan J, et al. Developing a framework for a novel multi-disciplinary, multi-agency intervention (s), to improve medication management in community-dwelling older people on complex medication regimens (MEMORABLE)—a realist synthesis. Systematic reviews. 2017; 6: 125.

For peer review only - http://bmjopen.bmj.com/site/about/guidelines.xhtml

16. Poland F, Mapes S, Pinnock H, et al. Perspectives of carers on medication management in dementia: lessons from collaboratively developing a research proposal. BMC Res Notes. 2014; 7: 463.
17. Mickelson RS, Holden RJ. Medication management strategies used by older adults with heart failure: a systems-based analysis. Eur J Cardiovascular Nurs. 2018; 17: 418-28.
18. Schafheutle EI, Fegan T, Ashcroft DM. Exploring medicines management by COPD patients and their social networks after hospital discharge. Int J Clin Pharm. 2018; 40: 1019-29.
19. Silvestre J, Reddy A, De La Cruz M, et al. Frequency of unsafe storage, use, and disposal practices of opioids among cancer patients presenting to the emergency department. Palliative & supportive care. 2017; 15: 638-43.
20. Wilson E, Caswell G, Turner N, et al. Managing Medicines for Patients Dying at Home: A Review of Family Caregivers' Experiences. Journal of Pain and Symptom Management. 2018; 56: 962-74.
21. Renny MH, Thaker RH, Dayan PS. Frequency of and Factors Associated With Prescription Medication Sharing Within Families. Pediatric Emergency Care. 2019. doi: 10.1097/PEC.0000000000001804
22. Markotic F, Jurisic D, Curkovic M, et al. Sharing of prescription analgesics amongst patients in family practice: Frequency and associated factors. Eur J Pain. 2018; 22: 716-27.
23. Kusturica MP, Sabo A, Tomic Z, et al. Storage and disposal of unused medications: knowledge, behavior, and attitudes among Serbian people. Int J Clin Pharm. pharmacy. 2012; 34: 604-10.
24. Hewson C, Shen CC, Strachan C, et al. Personal medicines storage in New Zealand. J Primary Health Care. 2013; 5: 146-50.
25. VanDyke MM, Steffen AM. Medication saving behaviors of older adults: Scale developed to assess family caregiver perspectives. Clin Gerontologist. 2017; 40: 258-67.
26. Brown MT, Bussell JK. Medication adherence: WHO cares? Mayo Clin Proc. 2011; 86: 304-14.
27. Pound P, Britten N, Morgan M, et al. Resisting medicines: a synthesis of qualitative studies of medicine taking. Soc Sci & Med. 2005; 61: 133-55.
28. McCoy L. Time, self and the medication day: a closer look at the everyday work of 'adherence'. Sociology of health & illness. 2009; 31: 128-46.
29. Townsend A, Hunt K, Wyke S. Managing multiple morbidity in mid-life: a qualitative study of attitudes to drug use. BMJ. 2003; 327: 837.
30. Dew K, Chamberlain K, Hodgetts D, et al. Home as a hybrid centre of medication practice. Sociology of Health & Illness. 2014; 36: 28-43.
31. Bali S, Stewart KA, Pate MA. Long shadow of fear in an epidemic: fearonomic effects of Ebola on the private sector in Nigeria. BMJ Global Health. 2016; 1: e000111.
32. Gasink LB, Linkin DR, Fishman NO, et al. Stockpiling drugs for an avian influenza outbreak: examining the surge in oseltamivir prescriptions during heightened media coverage of the potential for a worldwide pandemic. Infection Control & Hospital Epidemiology. 2009; 30: 370-76.
33. Lovegrove MC, Shehab N, Hales CM, et al. Emergency department visits for antiviral adverse events during the 2009 H1N1 influenza pandemic. Public Health Reports. 2011; 126: 312-17.
34. McLean KE, Abramowitz SA, Ball JD, et al. Community-based reports of morbidity, mortality, and health-seeking behaviours in four Monrovia communities during the West African Ebola epidemic. Global Public Health. 2018; 13: 528-44.
35. Basger BJ, Moles RJ, Chen TF. Application of drug-related problem (DRP) classification systems: a review of the literature. Eur J Clin Pharm. 2014; 70: 799-815.
36. Carson-Stevens A, Donaldson L, Sheikh A. The Rise of Patient Safety-II: Should We Give Up Hope on Safety-I and Extracting Value From Patient Safety Incidents?: Comment on" False Dawns and New Horizons in Patient Safety Research and Practice". Int J Health Policy & Management. 2018; 7: 667.

For peer review only - http://bmjopen.bmj.com/site/about/guidelines.xhtml

37. Abebe E, Scanlon MC, Lee KJ, et al. What do family caregivers do when managing medications for their children with medical complexity? Applied Ergonomics. 2020; 87: 103108.
38. Furniss D, Mayer A, Franklin BD, et al. Exploring structure, agency and performance variability in everyday safety: An ethnographic study of practices around infusion devices using distributed cognition. Safety Science. 2019; 118: 687-701.
39. Parand A, Faiella G, Franklin BD, et al. A prospective risk assessment of informal carers' medication administration errors within the domiciliary setting. Ergonomics. 2018; 61: 104-21.
40. Department of Health & Social Care and Public Health England. Guidance on shielding and protecting people who are clinically extremely vulnerable from COVID-19. Available at https://www.gov.uk/government/publications/guidance-on-shielding-and-protecting-extremely-vulnerable-persons-from-covid-19/guidance-on-shielding-and-protecting-extremely-vulnerable-persons-from-covid-19#Clinically Accessed 2020-10-14
41. Department of Health, Ireland. Guidance on cocooning to protect people over 70 years and those extremely medically vulnerable from COVID-19. Available at https://www.gov.ie/en/publication/923825-guidance-on-cocooning-to-protect-people-over-70-years-and-those-extr/ Accessed 2020-10-14
42. Pope C, Mays N. Synthesising qualitative research. Qualitative Research in Health care. 2006; 142: 152.
43. Bowen GA. Grounded theory and sensitizing concepts. Int J Qual Methods. 2006; 5: 12-23.
44. O'Brien BC, Harris IB, Beckman TJ, et al. Standards for reporting qualitative research: a synthesis of recommendations. Academic Medicine. 2014; 89: 1245-51.
45. Furniss D, Iacovides I, Lyons I, et al. Patient and public involvement in patient safety research: a workshop to review patient information, minimise psychological risk and inform research. Research Involvement and Engagement. 2016; 2: 19.
46. Garfield S, Jheeta S, Husson F, et al. Lay involvement in the analysis of qualitative data in health services research: a descriptive study. Research Involvement and Engagement. 2016; 2: 29.
47. Organization WH, Preparedness GRCfID. 2019 Novel Coronavirus Global Research and Innovation Forum: Towards a Research Roadmap Geneva, Switzerland: World Health Organization, 2020.
48. Sheikh A, Rudan I, Cresswell K, et al. Agreeing on global research priorities for medication safety: an international prioritisation exercise. J Global Health. 2019; 9.
49. Chang AY, Skirbekk VF, Tyrovolas S, et al. Measuring population ageing: an analysis of the Global Burden of Disease Study 2017. The Lancet Public Health. 2019; 4: e159-e67.
50. Slaintecare Report. Houses of the Oireachtas Committee, 2017. Dublin, Ireland. https://www.gov.ie/en/publication/0d2d60-slaintecare-publications/#the-slaintecare-report Accessed 2020-07-14.
51. National Institute for Health and Care Excellence. NICE Guidelines 67 Managing medicines for adults receiving social care in the community., 2017. https://www.nice.org.uk/guidance/ng67 Accessed 2020-07-14.

For peer review only - http://bmjopen.bmj.com/site/about/guidelines.xhtml

SRQR Checklist

Item	Comments for protocol	Comments for full manuscript
Item 1. Title: Concise description of the nature and topic of the study. Identifying the study as qualitative or indicating the approach (e.g., ethnography, grounded theory) or data collection methods (e.g., interview, focus group) is recommended.	Done, title, page 1	Same
Item 2. Abstract: Summary of key elements of the study using the abstract format of the intended publication; typically includes background, purpose, methods, results, and conclusions.	Done, abstract, page 1	
Item 3. Problem Formulation: Description and significance of the problem/phenomenon studied; review of relevant theory and empirical work; problem statement.	Problem statement, current need for this research, pages 3-4	
Item 4. Purpose or research question: Purpose of the study and specific objectives or questions.	Current need for this research, pages 3-4	
Item 5. Qualitative approach and research paradigm: Qualitative approach (e.g., ethnography, grounded theory, case study, phenomenology, narrative research) and guiding theory if appropriate; identifying the research paradigm (e.g., post-positivist, constructivist/interpretivist) is also recommended; rationale.	Described in methods, pages 4-5	
Item 6. Researcher characteristics and reflexivity: Researchers' characteristics that may influence the research, including personal attributes, qualifications/experience, relationship with participants, assumptions, and/or presuppositions; potential or actual interaction between researchers' characteristics and the research questions, approach, methods, results and/or transferability.	Described in methods, page 4	

For peer review only

Item 7. Context: Setting/site and salient contextual factors; rationale.	Methods, pages 4-5	
Item 8. Sampling strategy: How and why research participants, documents, or events were selected; criteria for deciding when no further sampling was necessary (e.g., sampling saturation); rationale.	Methods, page 5	
Item 9. Ethical issues pertaining to human subjects: Documentation of approval by an appropriate ethics review board and participant consent, or explanation for lack thereof; other confidentiality and data security issues.	Methods, page 7	
Item 10. Data collection methods: Types of data collected; details of data collection procedures including (as appropriate) start and stop dates of data collection and analysis, iterative process, triangulation of sources/methods, and modification of procedures in response to evolving study findings; rationale.	Methods, pages 5-7	
Item 11. Data collection instruments and technologies: Description of instruments (e.g., interview guides, questionnaires) and devices (e.g., audio recorders) used for data collection; if/how the instrument(s) changed over the course of the study.	Methods, page 6	
Item 12. Units of study: Number and relevant characteristics of participants, documents, or events included in the study; level of participation.	Methods, page 6-7	
Item 13. Data processing: Methods for processing data prior to and during analysis, including transcription, data entry, data management and security, verification of data integrity, data coding and anonymization / de-identification of excerpts.	Methods, page 6	
Item 14. Data analysis: Process by which inferences, themes, etc. were identified and developed, including	Methods, pages 6-7	

For peer review only

the researchers involved in data analysis; usually references a specific paradigm or approach; rationale.		
Item 15. Techniques to enhance trustworthiness: Techniques to enhance trustworthiness and credibility of data analysis, (e.g., member checking, triangulation, audit trail); rationale.	Methods, page 7	
Item 16. Synthesis and interpretation: Main findings (e.g., interpretations, inferences, and themes); might include development of a theory or model, or integration with prior research or theory.	Methods, within analysis, pages 6-7	
Item 17. Links to empirical data: Evidence (e.g., quotes, field notes, text excerpts, photographs) to substantiate analytic findings.	N/A at protocol stage	
Item 18. Integration with prior work, implications, transferability, and contribution(s) to the field: Short summary of main findings, explanation of how findings and conclusions connect to, support, elaborate on, or challenge conclusions of earlier scholarship; discussion of scope of application/generalizability; identification of unique contribution(s) to scholarship in a discipline or field.	N/A at protocol stage	
Item 19. Limitations: Trustworthiness and limitations of findings.	N/A at protocol stage	
Item 20. Conflicts of interest: Potential sources of influence or perceived influence on study conduct and conclusions; how these were managed.	Page 9	
Item 21. Funding: Sources of funding and other support; role of funders in data collection, interpretation, and reporting.	Page 8	

For peer review only
